# [Corrigendum] Overexpression of IL-9 induced by STAT3 phosphorylation is mediated by miR-155 and miR-21 in chronic lymphocytic leukemia

**DOI:** 10.3892/or.2025.8940

**Published:** 2025-07-02

**Authors:** Na Chen, Lili Feng, Huiting Qu, Kang Lu, Peipei Li, Xiao Lv, Xin Wang

Oncol Rep 39: 3064–3072, 2018; DOI: 10.3892/or.2018.6367

Following the publication of the above article, an interested reader drew to the authors' attention that, in Fig. 1A on p. 3067, the western blots shown for the STAT3 data in the upper right-hand gels (for the N3, N4, and C5-C8 experiments) were strikingly similar to the GAPDH data shown in the lower right-hand gels (for the C14-C16 and N6-N8 experiments). Furthermore, in Fig. 2A on p. 3068, the phosphorylated (p-)STAT western blots had apparently already appeared in a paper published by the same research group in the journal *International Journal of Clinical and Experimental Pathology.*

After inspecting their original data, the authors realized that the data of interest in Figs. 1 and 2 had inadvertently been chosen incorrectly. The revised and corrected versions of Figs. 1 and 2, now showing replacement data for the upper and lower right-hand gels in Fig. 1A and the upper three left-hand panels in Fig. 2A, are shown on the next two pages. Note that the errors made with the assembly of the data in these figures did not affect the overall conclusions reported in the paper. The authors apologize to the Editor of *Oncology Reports* and to the readership for any inconvenience caused.

## Figures and Tables

**Figure 1.**
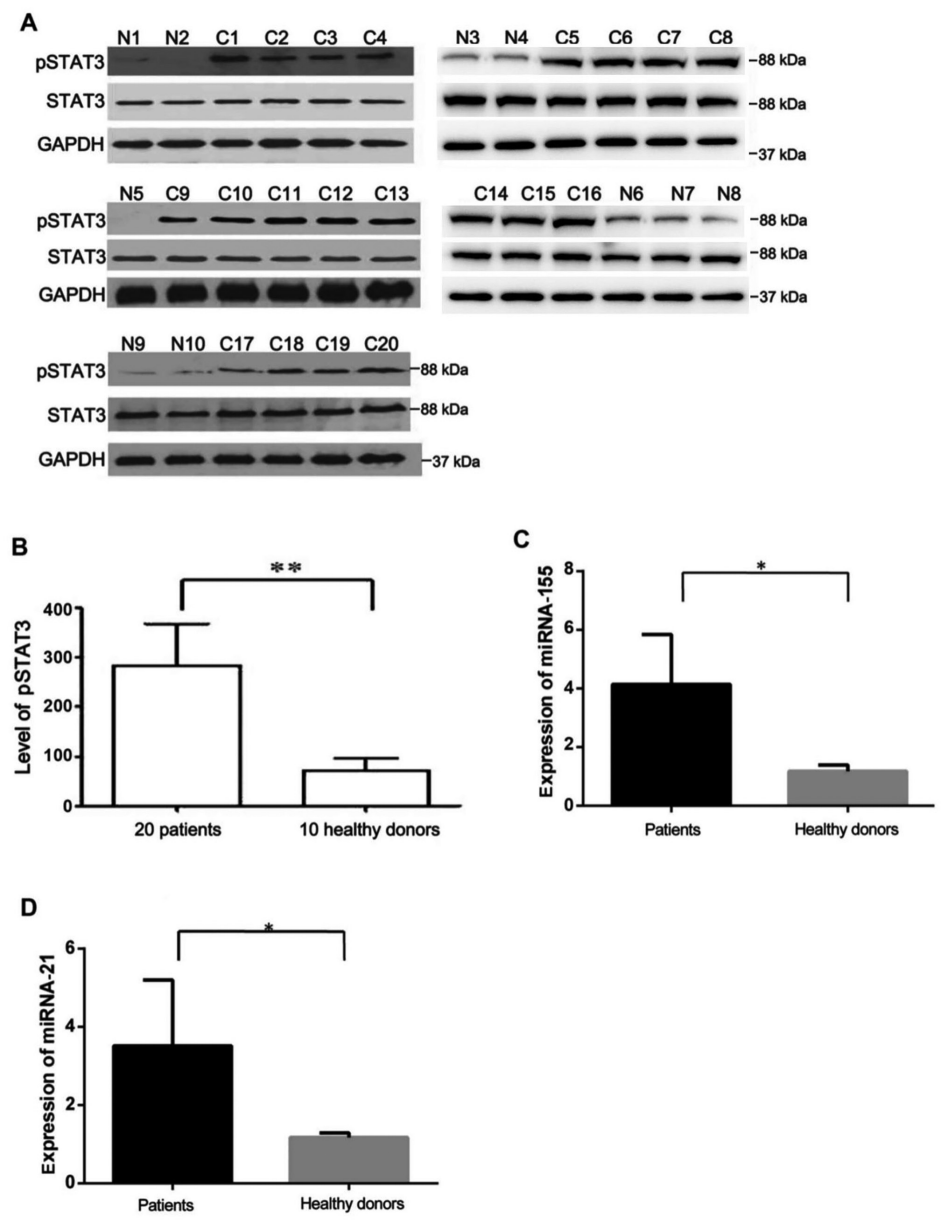
Expression of pSTAT3 protein and miRNAs in CLL patients and healthy controls. (A) The expression of the pSTAT3 protein was detected in 20 samples with upregulation of IL-9 (C), but none was detected in 10 PBMC from normal controls (N). (B) Relative density analysis of pSTAT3 protein bands. **P<0.0001. (C) RT-qPCR analysis of miR-155 expression in CLL patients and healthy controls. *P=0.0308. (D) RT-qPCR analysis of miR-21 expression in patient samples and healthy donors. *P=0.0398.

**Figure 2.**
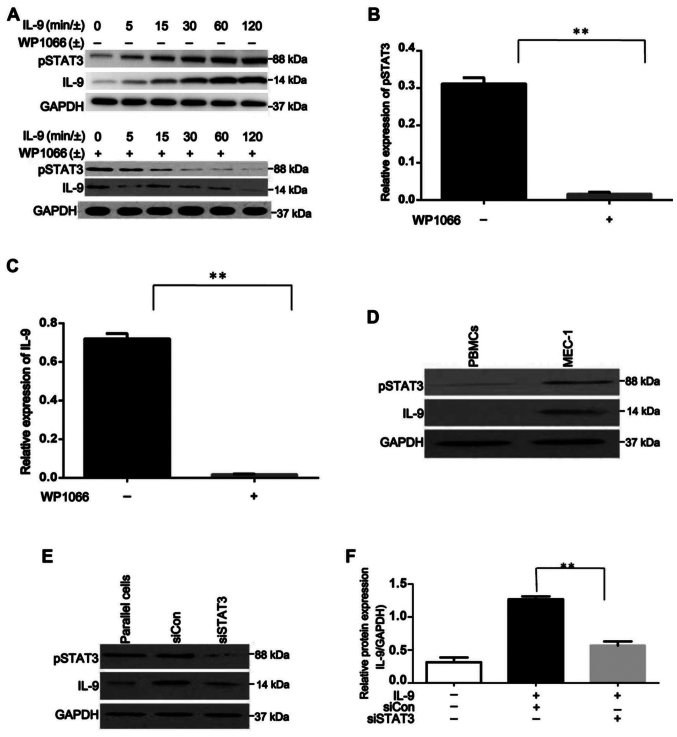
Effects of rIL-9 on pSTAT3 and IL-9 production in MEC-1 cells. (A) MEC-1 cells were pretreated with or without WP1066 for 48 h and then were incubated with rIL-9 for different times. STAT3 phosphorylation and IL-9 production in MEC-1 cells increased with the stimulation of rIL-9 in a time-dependent manner. WP1066, the STAT3 inhibitor, could markedly suppress the effects of rIL-9 on MEC-1 cells. (B) Relative density analysis of pSTAT3 protein bands, which were cultured with rIL-9 for 120 min. **P<0.0001. (C) Relative density analysis of IL-9 protein bands, which were treated with rIL-9 for 120 min. **P<0.0001. (D) Expression of pSTAT3 and IL-9 were detected by western blotting, with the single band size of 88 and 14 kDa, in peripheral blood mononuclear cells (PBMCs) from healthy samples and human CLL cell line MEC-1 after co-culture with rIL-9 for 120 min. (E and F) STAT3-knockdown cells exhibited significantly lower IL-9 expression in comparison to the cells transfected with negative control siRNA after co-culture with rIL-9 for 120 min. **P<0.0001.

